# Gene stacking in plant cell using recombinases for gene integration and nucleases for marker gene deletion

**DOI:** 10.1186/s12896-015-0212-2

**Published:** 2015-10-09

**Authors:** Soumen Nandy, Shan Zhao, Bhuvan P Pathak, Muthusamy Manoharan, Vibha Srivastava

**Affiliations:** Department of Crop, Soil & Environmental Science, 115 Plant Science Building, University of Arkansas, Fayetteville, AR 72701 USA; Department of Agriculture, 144 Woodard Hall, University of Arkansas at Pine Bluff, Pine Bluff, AR 71601 USA

**Keywords:** Gene stacking, Multigene transformation, Site-specific recombination, Targeted gene integration, Cre-*lox*, FLP-*FRT*, I-*Sce*I, ZFN

## Abstract

**Background:**

Practical approaches for multigene transformation and gene stacking are extremely important for engineering complex traits and adding new traits in transgenic crops. Trait deployment by gene stacking would greatly simplify downstream plant breeding and trait introgression into cultivars. Gene stacking into pre-determined genomic sites depends on mechanisms of targeted DNA integration and recycling of selectable marker genes. Targeted integrations into chromosomal breaks, created by nucleases, require large transformation efforts. Recombinases such as Cre-*lox*, on the other hand, efficiently drive site-specific integrations in plants. However, the reversibility of Cre-*lox* recombination, due to the incorporation of two *cis*-positioned *lox* sites, presents a major bottleneck in its application in gene stacking. Here, we describe a strategy of resolving this bottleneck through excision of one of the *cis*-positioned *lox*, embedded in the marker gene, by nuclease activity.

**Methods:**

All transgenic lines were developed by particle bombardment of rice callus with plasmid constructs. Standard molecular approach was used for building the constructs. Transgene loci were analyzed by PCR, Southern hybridization, and DNA sequencing.

**Results:**

We developed a highly efficient gene stacking method by utilizing powerful recombinases such as Cre-*lox* and FLP-*FRT*, for site-specific gene integrations, and nucleases for marker gene excisions. We generated Cre-mediated site-specific integration locus in rice and showed excision of marker gene by I-*Sce*I at ~20 % efficiency, seamlessly connecting genes in the locus. Next, we showed ZFN could be used for marker excision, and the locus can be targeted again by recombinases. Hence, we extended the power of recombinases to gene stacking application in plants. Finally, we show that heat-inducible I-*Sce*I is also suitable for marker excision, and therefore could serve as an important tool in streamlining this gene stacking platform.

**Conclusions:**

A practical approach for gene stacking in plant cell was developed that allows targeted gene insertions through rounds of transformation, a method needed for introducing new traits into transgenic lines for their rapid deployment in the field. By using Cre-*lox*, a powerful site-specific recombination system, this method greatly improves gene stacking efficiency, and through the application of nucleases develops marker-free, seamless stack of genes at pre-determined chromosomal sites.

## Background

Practical approaches for gene stacking are a critical need of crop biotechnology as the future requires multigene engineering for expressing complex traits, and periodic introduction of new traits into the previously engineered crops. The conventional methods of plant transformation integrate genes into undetermined chromosomal sites. As a result, breeding of transgenes into a single line becomes exponentially complex with the increase of transgenic donor lines or the number of independently segregating transgenes. To avoid segregation of transgenes, methods for targeted gene integration into the pre-determined genomic sites or gene stacking are needed. Gene stacking can resolve the complexity of multi-trait breeding, and ensure timely deployment of new traits into diverse crop varieties [1]. Targeted gene integration can be practiced by two distinct approaches, homologous (and non-homologous) recombination into the chromosomal double-stranded breaks (DSB) induced by synthetic or rare nucleases [2–4], and site-specific recombination (SSR) into the pre-integrated recombination sites [5–7]. Since the repair of DSBs could occur through one of the multiple pathways [8], precise targeted gene integration is practically a rare outcome, requiring development and screening of a high number of transgenic clones [3, 4]. SSR, on the other hand, is a simple, predictable reaction leading to a high number of transgenic clones that contain precise site-specific integration [9–13].

A number of SSR systems are known, Cre-*lox* among them stands out for its high efficiency in complex genome engineering in eukaryotic cells including plants [14–17]. So far, four SSR systems have been successfully used for directing site-specific integration (SSI) in plants, that include Cre-*lox*, FLP-*FRT*, R-*RS*, and Bxb1 [9, 12, 18, 19]. The efficiency of Cre-*lox* and FLP-*FRT* combined with the strategy to select SSI clones greatly improves the transformation pipeline through high SSI recovery rates and enhanced production of stable lines [13, 20–22]. However, Cre-*lox* and FLP-*FRT* recombination are reversible as they generate two recombination sites (*loxP* or *FRT*) in SSI (*cis*-positioned) that can recombine to reverse the structure. While, mechanisms of controlling the reversibility of recombination are highly effective [9, 10, 13, 23], they are not suitable for the iterative applications. Previously, we developed a selection marker gene (SMG)-free site-specific integration approach based on the use of FLP-*FRT* for gene integration followed by the use of Cre-*lox* for SMG-deletion [24]. This approach generated marker-free lines in a single generation that transmitted the marker-free SSI to the progeny at >95 % efficiency. While, this approach is suitable for integrating DNA constructs containing multiple genes, it cannot easily be used for the repeated rounds of transformation that would be needed for adding new traits to the ‘stacked site’.

Here, we modified the ‘marker-free site-specific integration’ approach by introducing nucleases for SMG deletion. Concomitant cuts on a chromosome could delete the intervening fragment, and the broken ends of the chromosome could join through DNA repair process. The repaired site incorporates sequence changes that are no longer targeted by the same nuclease [8]. Therefore, a given nuclease can be used again for SMG deletion. If one of the two *cis*-positioned *lox* or *FRT* is present within this fragment, it will also be deleted, leaving a single site for gene stacking. This modified method for marker-free site-specific integration is suitable for iterative gene stacking. Each of the steps in this method, individually, have been demonstrated by earlier studies. Cre or FLP-mediated site-specific gene integration, and nuclease mediated DNA deletion have all been demonstrated in plants [9–13, 25–28]. However, these diverse molecular mechanisms have not been integrated in a single platform. Where Cre-*lox* is well-known for generating highly precise recombination products [16], nuclease-induced DSB repair could create insertion-deletions (indels) at the site [8]. Therefore, for this gene stacking approach to be successful, it is important to establish that nuclease-induced indels would not alter the adjacent sequences, too often, or interfere with Cre-*lox* components in the iterative site-specific integrations. The focus of the present work is to develop the proof-of-concept for the proposed marker-free gene stacking strategy by characterizing the deletion sites induced by I-*Sce*I and *CCR5*-ZFN at the Cre-mediated SSI locus in rice, and demonstrate the second round of site-specific integration in the same site. We first generated SSI lines using a founder line, T5, and subjected them to SMG deletion by I-*Sce*I, and carried out a second round of site-specific integration by FLP-*FRT* and characterized *CCR5*-ZFN mediated DSB/repair sites. We also tested the utility of heat-inducible I-*Sce*I and ZFN in DNA deletions. Overall, this work shows that Cre-*lox* and FLP-*FRT* can be used for gene stacking in plants, and their power extended to iterative genome engineering applications.

## Results

### Molecular strategy

**Fig. 1:**
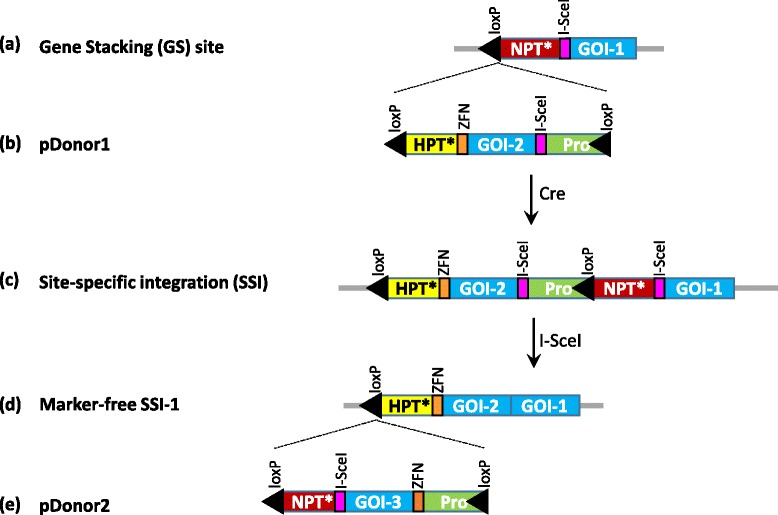
Gene stacking approach based on the use of Cre-*lox* for gene integration and a pair of nucleases for marker deletion. **a** Gene stacking site consists of a *loxP* fused to promoter-less marker gene (NPT*) followed by the first nuclease target site, e.g., I-*Sce*I site. The GS construct could also include the first set of gene(s)-of-interest (GOI-1); **b** The first donor vector, pDonor1, contains *loxP* flanked construct consisting of the second promoter-less marker gene (HPT*), the second nuclease target site, e.g., ZFN site, GOI-2, the first nuclease target site, and the promoter for activating *NPT* gene at the GS site; **c** Co-delivery of pDonor1 and *cre* gene generates site-specific integration (SSI) locus, which is selectable due to the activation of *NPT* gene, and contains two I-*Sce*I target sites for marker excision; **d** Delivery of I-*Sce*I gene into SSI cells leads to excision of *NPT* gene, seamlessly connecting GOI-1 and GOI-2; **e** The second donor vector, pDonor2, is similar to pDonor1 except the first marker gene, *NPT*, is included and nuclease target sites are exchanged. Targeting of pDonor2 into marker-free SSI-1 line will generate SSI-2 that is selectable due to *HPT* activation, and converted to marker-free SSI-2 by ZFN activity

The strategy of iterative gene stacking is based on the use of an efficient site-specific recombination (SSR) system and a pair of nucleases to direct transgene integration and selection marker gene (SMG) deletion, respectively (Fig. 1). We propose using Cre-*lox* or FLP-*FRT* for transgene integration for their robust activity and high specificity in plants [14, 15]. Cre or FLP-mediated gene integration, however, results in the incorporation of two *cis*-positioned *lox* or *FRT* sites (Fig. 1a-c), which can excise out the integrated DNA in the subsequent rounds of transformation. Therefore, SMG deletion is carried out by nuclease, which removes one of the two *cis*-positioned recombination sites along with the SMG fragment (Fig. 1d). The single recombination site in the SMG-free SSI site serves as the target for the next round of site-specific integration. It should be noted that SMG removal is an integral step in gene stacking, therefore, no extra effort is added in this strategy. We used efficient nucleases, I-*Sce*I and *CCR5*-ZFN, in this study; however, other nuclease technologies, if found to be efficient, can also be used. As shown in Fig. 1, the strategy utilizes promoter/gene trap to isolate site-specific integration events (Fig. 1a-c), which are subjected to nuclease-mediated marker deletion to generate marker-free SSI (Fig. 1d). This process could be repeated to achieve gene stacking into the selected chromosomal sites.

### Development of site-specific integration lines

**Fig. 2:**
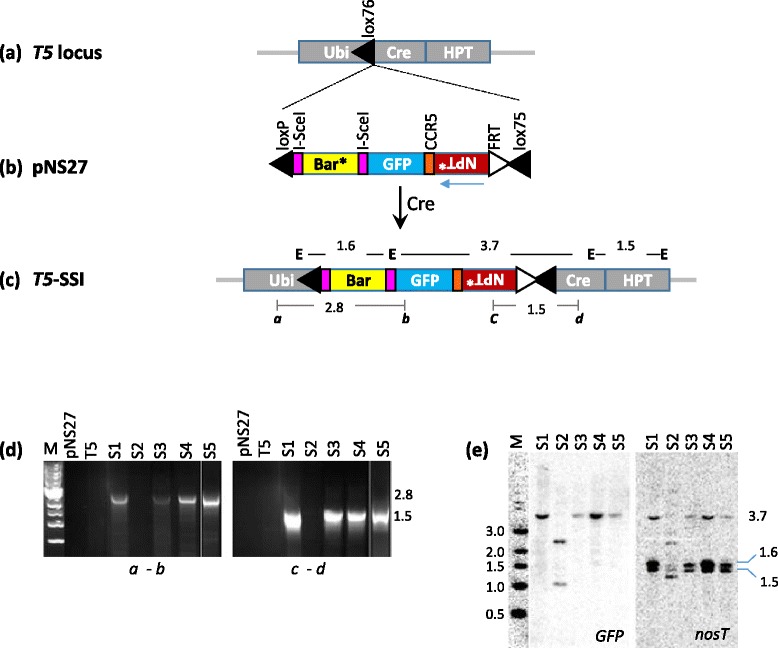
Site-specific integration into *T5* locus. The development of T5 line is described earlier [28]. **a** *T5* site contains *lox76* (right arm mutant) between maize ubiquitin-1 (Ubi) promoter and *cre* coding sequence, and a hygromycin selection marker (*HPT*); **b** Donor plasmid pNS27 (pUC backbone), designed for site-specific integration into *T5* site, contains between *loxP* (wild-type) and *lox75* (left arm mutant), a promoter-less *Bar* gene (*), *GFP* gene (35S:GFP), promoter-less *NPT* gene, and *FRT* site. *Bar* gene is flanked by I-*Sce*I target sites, and a single *CCR5* site is present downstream of *NPT* gene. Each gene contains *nos 3′* transcriptional termination (*nosT*) sequence (not shown); **c** Introduction of pNS27 into T5 cells, that already express Cre activity, results in the integration of the donor construct into *T5* site via *lox75* x *lox76* recombination, generating precise site-specific integration (SSI) that contains *loxP* downstream of Ubi, and double-mutant *lox78* upstream of *cre* (black triangles); **d** PCR analysis of *T5*-SSI lines (S1 – S5) using primers shown above; **d** Southern hybridization of *Eco*R1 (**e**) digested genomic DNA of SSI lines with GFP and nosT fragments. M, 1-kb DNA ladder. The expected PCR and *Eco*RI fragments from precise SSI site are shown in (**c**)

To develop SSI locus containing I-*Sce*I and ZFN target sites, pNS27 was developed for DNA integration into *T5* site, which contains a *lox76* site for Cre-mediated gene integration, and expresses Cre activity (Fig. 2a). pNS27 contains promoter-less *Bar* gene flanked by I-*Sce*I target sites, *GFP* gene, *CCR5* site (ZFN target), and promoter-less *NPT II* gene fused to *FRT* for FLP-mediated site-specific integration (Fig. 2b). Delivery of pNS27 into T5 calli generated 5 bialaphos-resistant SSI lines, S1 – S5, from ten bombarded calli plates. All except one (S2) were confirmed to contain the expected SSI structure by PCR, sequencing, and Southern hybridization (Fig. 2c-e; sequencing data not shown). Two of these (S1 and S4) were used for the subsequent work.

### I-*Sce*I-mediated marker deletion from SSI locus

**Fig. 3:**
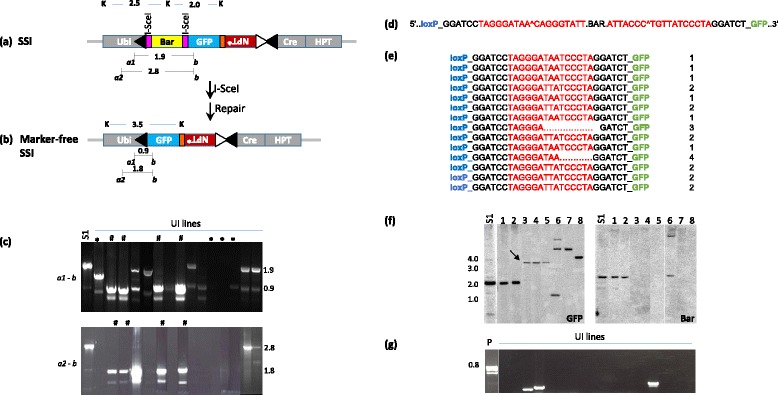
I-*Sce*I-mediated marker excision from site-specific integration (SSI) locus. **a** The structure of *T5*-SSI containing I-*Sce*I targets (magenta bars) around *Bar* gene; **b** Introduction of I-*Sce*I activity by retransformation of the SSI line, S1, excises *Bar* gene to generate marker-free SSI; **c** PCR on UI lines (S1 line retransformed with Ubi:ISceI construct) amplifies ‘excision’ footprints (0.9 or 1.8 kb), indicative of precise excision (#) or indels (*); **d** Sequence of SSI locus around *Bar* gene containing *loxP* upstream and *GFP* downstream. Red fonts represent I-*Sce*I targets (nick site indicated by ^); **e** Sequences of the 0.9 kb excision ‘footprints’ obtained from UI lines. Concomitant DSB on the two I-*Sce*I sites followed by joining generates near-perfect repair without the loss of sequences immediately adjacent to the I-*Sce*I targets. The numbers indicate the four repair sequences (1–4) observed in perfect excision lines; **f** Southern blot analysis of UI lines on *Kpn*I (K)-digested genomic DNA using *GFP* and *Bar* probe. Arrow points out the expected excision band (3.5 kb) from near-perfect excision locus. S1, parental SSI line; **g** PCR analysis of UI lines to detect the presence of Ubi:ISceI gene. Note the absence of the expected 0.8 kb band in UI lines. P, positive control (pUbi:ISceI)

S1 and S4 were retransformed by co-bombardment with pUb:ISceI and pSS1 (the selection plasmid containing 35S:NPT gene), and the calli were selected on geneticin. The resistant lines were analyzed for the deletion of *Bar* gene from the SSI locus. A total of 72 geneticin resistant callus lines (UI) were isolated, each of which was subjected to PCR using primers around the *Bar* gene (Fig. 3a-b). The concomitant DSBs created by I-*Sce*I are expected to delete the *Bar* gene, and the broken ends would be repaired through the DNA repair process. Since homologous DNA sequences are not present at the broken ends, repair through non-homologous end joining mechanism would occur that incorporates insertions – deletions (indels) at the repaired site [8]. The uncut SSI locus is expected to generate 1.9 and 2.8 kb amplicons with primers *a1*-*b* and *a2*-*b*; however, precise deletion of *Bar* would reduce the amplicon size to 0.9 and 1.8, respectively (Fig. 3a-b). Fourteen UI lines (19.4 %) showed 0.9 and 1.8 kb bands without the presence of the parental bands in PCR with *a1* - *b* and *a2 - b*, indicating near-perfect repair of the cut site (Fig. 3c; Table 1). Sequencing of 0.9 kb fragments showed that the excision-repair in these lines occurred without the loss of sequences immediately adjacent to the I-*Sce*I sites, confirming near-perfect repair of the site (Fig. 3d-e). These 14 lines contained 4 types (1 – 4) of the repaired sequences, with type 1 and 2 found frequently, and type 3 and 4 found in one line each (Fig. 3e). Specifically, 12 lines contained either type 1 (TAGGGATAATCCCTA) or type 2 (TAGGGATTATCCCTA) sequence that differed in the *5′*-overhang (*5′*-ATAA) generated by the I-*Sce*I cut. Since the overhangs from the two sites (upstream and downstream of *Bar*; Fig. 3d) are complementary in 2 of 4 bases, they could initiate microhomology based recombination [29, 30]. Type 3 and 4, on the other hand, contain very short deletions (≤10 bases) at the repair site. The rest of the lines either contained no change or imperfect deletion (Fig. 3c; Table 1). Seven lines (9.7 %) contained short indels at the excision site, indicated by amplification of bands larger or smaller than 0.9 or 1.8 kb. Twenty five lines (48.6 %) did not amplify any product, indicating the presence of large indels at the excision site, and 26 lines (36 %) amplified the parental 1.9 and/or 2.8 kb band expected from the intact SSI site (no change). While, large indels could not be analyzed due to the lack of PCR amplification, analysis of short indels showed that DSB sites frequently incorporated vector DNA sequences, while one line contained rice mitochondrial DNA (data not shown).

**Table 1:** I-*Sce*I and ZFN activity at the site-specific integration locus

Lines	Nuclease	Expression system^1^	Total #	Perfect deletion^2^	Short indels^3^	Large indels^4^	No change^5^
UI	I-*Sce*I	Constitutive	72	14	7	25	26
UZ	ZFN	Constitutive	68	4	5	38	21
HI	I-*Sce*I	Heat-shock	7	3	-	-	4
HZ	ZFN	Heat-shock	23	-	-	13	10

^1^Strong constitutive (Ubi promoter) or heat-shock (HS) expression

^2^ < 15 bp indel at the target without the loss of sequences immediately adjacent to the nuclease target

^3^50–200 bp insertion or deletion

^4^No amplicons obtained in PCR

^5^No DSB-repair as indicated by PCR and/or sequencing

Southern blot analysis was done on the genomic DNA isolated from the regenerated plants of the UI lines. Probing of the *Kpn*I-digested genomic DNA with *GFP* fragment generated the expected ~2 kb band from S1 parent, which upon deletion of *Bar* fragment that contains a *Kpn*I site is expected to increase to 3.5 kb (Fig. 3a-b). Southern data corroborated with the PCR data as the UI lines that were found to contain the near-perfect deletion in PCR showed 3.5 kb band upon hybridization with *GFP*, and those having imperfect deletions (short/large indels) showed variable sizes, while the lines found to contain the parental locus showed the intact 2 kb band (Fig. 3f). Re-probing this blot with the *Bar* fragment confirmed complete deletion of *Bar* gene from the ‘near-perfect’ UI lines (Fig. 3f). In the PCR to amplify I-*Sce*I gene, surprisingly, none of the UI lines were found to contain full I-*Sce*I construct as indicated by the absence the expected 0.8 kb amplicon or the presence of smaller amplicons indicating truncated/rearranged I-*Sce*I fragments (Fig. 3g). The exclusion of the functional I-*Sce*I gene in UI lines points to the potential toxicity of the strong constitutive I-*Sce*I activity in rice. Therefore, deletion of *Bar* gene in rice cells essentially occurred by the transient I-*Sce*I expression. This data also explains the failure of *Bar* deletion in a large proportion of UI lines (36 %). A BLAST search for I-*Sce*I target site identity in rice genome found partial matches with 14 loci, in which 10–15 bases out of 18 base I-*Sce*I sequence (≤70 %) matched. Two of these loci that showed 15 and 13 base match on chromosome 10 were analyzed. PCR/sequencing of these sites (NC_008403.2|:22808202–22809528 and NC_008403.2|:20914800–20915400) in 6 different UI lines, three of which contained perfect *Bar* deletion, found no sequence change (Data not shown). In summary, I-*Sce*I mediated marker deletion was found to occur in 46 of 72 lines (63 %), 14 of which (19.4 %) showed perfect deletion without the loss of the adjacent sequences at the SSI locus. Marker-deletion in the SSI lines essentially occurred by the transient I-*Sce*I activity as strong constitutive expression appeared to be toxic to rice genome.

### Subsequent rounds of site-specific integration by FLP-*FRT* system

**Fig. 4:**
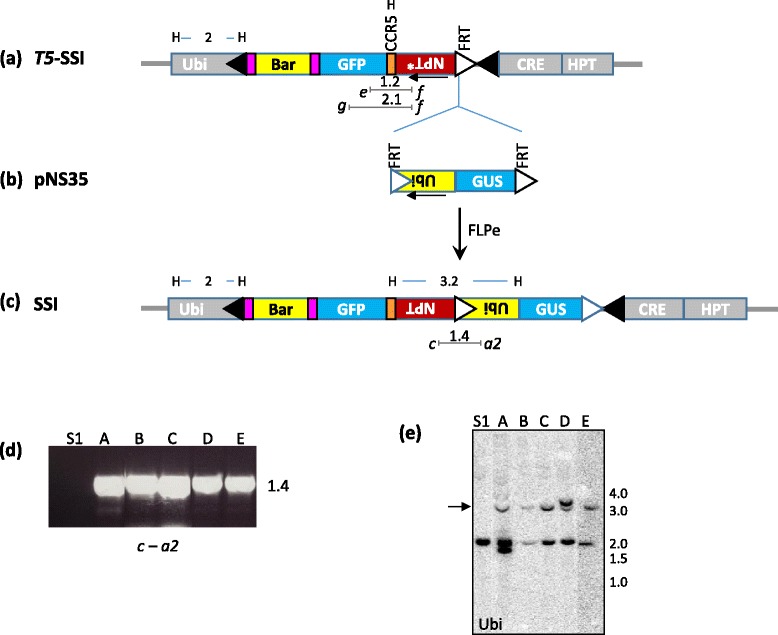
Site-specific integration into *T5-*SSI by FLP-*FRT* recombination. **a** Structure of the *T5*-SSI obtained through Cre-mediated site-specific integration (see Fig. 2), note the presence of promoter-less (*) *NPT* gene and *FRT* site for the next round of site-specific integration; **b** Donor vector, pNS35, contains *FRT* flanked maize ubiquitin promoter (Ubi) oriented to trap *NPT* gene in the marker-free SSI site, and the *gus* gene as gene-of-interest; **c** Delivery of pNS35 and FLPe gene generates SSI locus through *FRT x FRT* recombination. This SSI is selectable due to the activation of *NPT* gene; **d** PCR analysis of geneticin resistant SSI lines showing the presence of the expected 1.4 kb SSI footprint; **e** Southern hybridization of SSI lines (A – E) on *Hind*III-digested genomic DNA probed with Ubi fragment. *Hind*III (H) sites, fragment sizes, primer sites are shown in the SSI structure (c). DNA sizes are shown in kb

The gene stacking strategy shown in Fig. 1 can be practiced by a single SSR system. However, *T5* locus used in the present work is not designed for iterative gene stacking, therefore, we included an *FRT* site in pNS27 construct for FLP-mediated site-specific integrations into marker-free SSI lines. The *T5*-SSI locus contains a promoter-less *NPT* gene fused to *FRT* site. Therefore, site-specific integration into *FRT* site will be selectable through *NPT* activation. This strategy of promoter - gene fusion based selection in which an *FRT* site is located between the promoter and *NPT* gene (Fig. 4a-c) was demonstrated in our previous work [13]. Using the same strategy, we showed that the *FRT* located in the SSI site, e.g., S1 locus, can be targeted for gene stacking by subsequent transformation. A new donor vector, pNS35 that contains *FRT*-flanked Ubi promoter, was delivered by co-bombardment with pUbiFLPe into S1 line, and the geneticin-resistant lines were analyzed by PCR and Southern hybridization for the presence of SSI structure arising from *FRT* x *FRT* recombination (Fig. 4b-c). The use of FLPe is critical in obtaining site-specific integration as this variant of FLP is several fold more efficient in both DNA excisions and integrations [13, 31, 32]. From the bombardment of 10 plates, 5 SSI lines (A – E) were recovered that expressed *GFP* and *GUS* (data not shown), and contained the predicted 1.4 kb Ubi:NPT fusion in PCR with primers *c* and *a2* (Fig. 4c-d). Southern hybridization of the *Hind*III (H) digested DNA of these lines with Ubi probe showed the presence of the expected ~3.2 and 2 kb bands in each line, while the parent S1 line contained only 2 kb band. Two of the SSI lines (A and D) contained additional bands, while the remaining three contained only the expected bands, indicating the absence of extra-SSI fragments (Fig. 4e). Overall, this analysis confirmed the reactivity of the *FRT* site in the SSI locus and validated its suitability as the target for the next round of gene stacking.

### Testing ZFN activity in rice genome

**Fig. 5:**
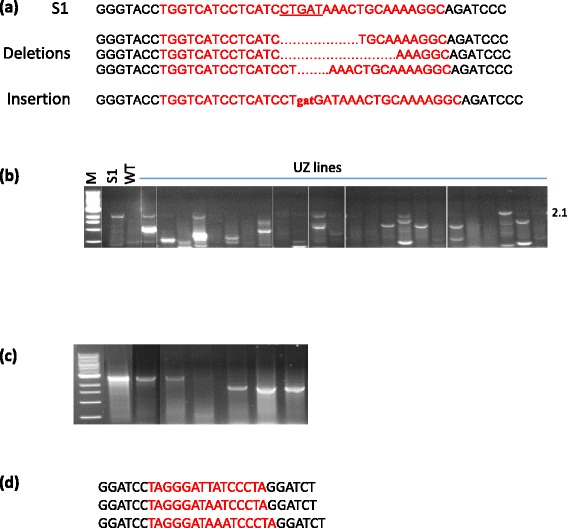
ZFN and heat-inducible I-*Sce*I activity. **a** Sequences of *CCR5* site amplified by PCR using *e – f* primers (see Fig. 4a). Sequences obtained from the parental S1 line and four UZ lines (transformed with pUbi:ZFN). Deletion is shown by dots and insertion by small case letters. Red fonts indicate *CCR5* sequence; **b** PCR analysis of UZ lines with *g – f* primers to detect large deletions at *CCR5* site. Note the presence of <2.1 kb bands in different lanes; **c** PCR analysis of HI lines (1–3, S1 line retransformed with heat-inducible I-*Sce*I gene). HI samples maintained at room temperature (RT) or exposed to heat-shock (HS) were analyzed to detect the excision of *Bar* gene using primers *a2* - *b* (see Fig. 3a). Note the presence of 1.8 kb excision footprint in HS samples; **d** Sequences of 1.8 kb excision footprints from HS samples. Red fonts indicate I-*Sce*I target sequences

As shown in Fig. 1, two nucleases are required for the alternating use in the gene stacking process. The second nuclease tested in this study is ZFN that targets human *CCR5* sequence [33]. A single *CCR5* site consisting of 33 nucleotides is located in the SSI locus (Fig. 5a). To test the activity of ZFN at this site, S1 line was retransformed with pUbi:ZFN, and the transformed callus lines (UZ lines) were analyzed by PCR using primers *e* and *f* (Fig. 4a). This PCR is expected to generate 1.2 kb amplicon from the SSI locus, and upon DSB/repair at *CCR5* site, could either generate the same size amplicon (very short indels or no change), variable size amplicon (short indels) or no amplicon (large indels). A total of 68 UZ lines were analyzed, 38 of which (55.8 %) did not generate any amplicon, indicating the presence of large indels. The sequencing of the amplicons obtained (sizes ranging from 0.8 to 1.1 kb) from the remaining 30 lines, showed that 21 lines contained no sequence change, and nine contained very short or short indels at the *CCR5* site (30 %) (Table 1). Four of these lines contained a very short insertion or deletion, with three lines containing only 3–14 bp deletion and 1 line containing 3 bp insertion (Fig. 5a). Overall, this analysis indicated that *CCR5*-ZFN efficiently creates DSB at the *CCR5* site placed in the rice genome, as 47 out of 68 lines (69 %) contained indels at the *CCR5* site. However, most of these lines contained large indels and possibly incorporated sequences of the introduced plasmid DNA. To distinguish between large deletions and insertions, PCR on 24 of these lines was conducted using primers *g* and *f* that is expected to amplify 2.1 kb fragment from the SSI locus (Fig. 4a). Thirteen of these lines amplified fragments of variable sizes, indicating large (>1 kb) deletions at the *CCR5* site, since the analysis was done on callus, which could consist of more than one cell line, most of these samples also generated the parental 2.1 kb band (Fig. 5b). Although insertion was observed frequently in this study, most likely it occurred due to the presence of high quantities of exogenous DNA in the form of the introduced plasmids. Finally, the integration of *ZFN* gene in these lines was tested by PCR, which showed that the majority of the lines (46 out of the 68) contained *ZFN* gene, however, a fair number of lines (22 lines) did not contain *ZFN* gene, in spite of showing indels at the *CCR5* site. Therefore, transient ZFN activity could also induce DSB at *CCR5* site in the rice genome. It should be noted that a relatively small quantity of Ubi:ZFN plasmid was bombarded (100 ng per shot), which could explain a high proportion of lines lacking stable *ZFN* integration.

### Testing heat-inducible nuclease activity for marker deletion

The nuclease activity can be introduced into SSI lines by retransformation, genetic crosses or inducing gene activity. The use of inducible nucleases is particularly attractive for reducing effort in the process. Previously, we showed that heat-inducible Cre or FLPe are effective in excising SMG from rice by exposing seedlings to 42 °C for 3 h [24, 34]. Here, we assessed the efficacy of heat-inducible I-*Sce*I and ZFN in deleting SMG from the *T5*-SSI locus (Fig. 2c). HS:ISceI or HS:ZFN constructs were introduced into S1 line by retransformation, and the resulting clones (HI or HZ) were confirmed by PCR to contain the respective nuclease gene (Data not shown). Calli of 7 HI and 23 HZ lines were incubated at 42 °C for 3 h, transferred to room temperature for 72 h, and ground for DNA extraction. Due to the presence of two I-*Sce*I targets, I-*Sce*I activity could be detected by PCR using *a2-b* primers (Fig. 3a-b), whereas, ZFN activity can only be detected by sequencing the PCR product as only one ZFN target (*CCR5*) is present (Fig. 4a). Three out of 7 HI lines generated 1.8 kb deletion fragment with *a2 - b* primers in the heat-treated samples (Fig. 5c). Sequencing of these fragments showed near-perfect deletion of *Bar* gene (Fig. 5d), confirming the efficacy of heat-inducible I-*Sce*I. The remaining HI lines showed the parental 2.8 kb band in the heat-treated samples, indicating poor I-*Sce*I induction or gene silencing. PCR analysis of 23 heat-treated HZ lines with *e – f* primers (Fig. 4a) found 1.2 kb amplicon in 10 lines, <1.0 kb amplicon in 2 lines, and no amplification in 11 lines (data not shown), indicating large indels in the latter 13 lines. The sequencing of 1.2 kb amplicons of the former ten lines, however, did not show indels at the *CCR5* site in any of the lines. A number of these lines failed to amplify the expected 1.2 kb band even without heat-treatment, indicating promiscuous ZFN activity. Therefore, more work is needed to determine the efficiency of HS:ZFN for inducible marker excision in plants.

## Discussion

The current methods of multigene transformations include transfer of large T-DNA or co-transformation of multiple vectors [35, 36], which can deliver multiple genes in a single attempt but are not suitable for the periodic introduction of new genes into the engineered sites. Targeted gene integrations in plants by synthetic nucleases has been reported in recent years [37–42], the mechanism of which is the repair of chromosomal breaks through homologous recombination. However, since the repair could also occur by joining of the broken chromosomal ends through non-homologous recombination, targeted gene integrations are practically infrequent [4, 39]. Targeted integrations at 3–5 % efficiency has been reported in crop plants [3]; however, it is generally accepted that isolation of targeted events require large transformation efforts even when a selection strategy is employed to isolate targeted insertions [4, 42].

Efficient gene stacking approaches could become widely practiced technologies. The use of Cre-*lox* system, in combination with the selection strategies, is extremely successful in producing high quality SSI clones without significantly lowering plant transformation efficiencies [9, 11]. Similarly, FLP-*FRT* is effective in producing SSI clones at high efficiency in plants [12, 13]. These SSR systems are reliable as they have been widely used and found to be effective in a number of plant species [14]. However, the reversibility of recombination, especially the recombination leading to SSI, remained a major bottleneck in developing iterative site-specific gene integration approaches. The use of mutant *lox* sites for controlling reversibility would allow only two rounds of integrations as the recombination between a set of mutant *lox* sites generates a *loxP* [9], which can be targeted, but two *cis*-positioned *lox* sites will be generated in the resulting SSI that would complicate subsequent use of Cre. However, excising one of the two *cis*-positioned sites, along with the marker gene, resolves the bottleneck without increasing the effort, and allows, in principle, unlimited rounds of gene stacking. The use of nucleases for marker excision is of strategic importance as the cut-repair site cannot be targeted by the same nuclease. Recently, a number of studies have described the use of nucleases in targeted mutagenesis [43], demonstrating their effectiveness and efficiency in plants. Excision of marker gene involves two concomitant breaks to delete a precise segment of DNA. Two studies have demonstrated the use of nucleases in marker gene excision. Antunes et al. [25] found ~15 % efficiency of PB1 in Arabidopsis for marker excision and inheritance of the excision locus. Petolino et al. [27] used *CCR5*-ZFN to delete transgenes from maize, and reported high rate of excision (35 %) in F1 plants, several of which stably transmitted the excision locus to F2 progeny. For gene stacking application, it is important that the sequences beyond the nuclease target site are preserved, and the integrity of the locus is not compromised. Using heat-inducible PB1, Antunes et al. found a fair number of clones containing very short indels or perfect re-ligation of the cut ends. A similar result was obtained by Petolino et al. in examining the site that were targeted by ZFN. These reports along with our data confirm the practical application of nucleases in deleting marker genes from plants. The use of inducible nucleases could streamline marker excision process and allow marker excision in the primary transgenic plants. The use of inducible nucleases could also allay the concern of off-target DSBs as we found that rice cells could tolerate heat-inducible I-*Sce*I expression, but not the strong constitutive expression. Although, off-targeting by strong constitutive expression of I-*Sce*I has not been reported in plants, and we did not find off-targeting at the predicted genomic sites in rice, off-targeting at non-canonical sites has been reported in human genome [44].

The gene stacking strategy presented here can be practiced with the current transformation platforms using dedicated target lines and designed donor constructs. SSR-mediated gene integrations have been practiced with both *Agrobacterium* and particle bombardment methods [12, 13, 23, 28]. Nuclease-mediated marker excisions could be done by genetic crosses or by deploying inducible nucleases as part of the donor construct (Fig. 1b, e: placed next to the promoter-less marker gene, upstream of nuclease target site) and integrating it into SSI structure for controlled ‘auto-excision’, an approach that has been demonstrated with Cre-*lox* [24, 45, 46]. More work is needed to determine the efficiency of the auto-excision approach with nucleases. More importantly, the two nucleases used in this study were effective in creating DSB or deleting marker gene, and, in case of I-*Sce*I, preserving the integrity of the SSI locus. Hence, each step of the proposed gene stacking strategy was validated and found to be efficient, suggesting that this method is likely to function at 20 – 50 % efficiency in rice. Its efficiency in other plant species is also expected to be high as the efficiency of Cre-*lox* recombination and nucleases, individually, are reportedly high [27, 45, 47–49]; however species to species variation could occur, e.g., due to variation in DSB repair mechanisms [50].

Gene stacking methods that do not involve the use of nucleases have been reported. Two rounds of recombinase-mediated cassette exchange (RMCE) using 3 hetero-specific *FRT* sites was used for stacking 7 genes in soybean [51]. However, its application in subsequent rounds would be limited by the availability of new hetero-specific *FRT* sites. Currently, only a limited number of hetero-specific *FRT* sites are available, reactivity of which is variable [52]. Bxb1 system involves recombination between two non-identical sites (*attP* x *attB*), which cannot be reversed by Bxb1 recombinase without the helper protein [53]. This unidirectional recombination mechanism was used for gene stacking in tobacco through three rounds of transformation [19]. The resulting ‘stacked’ site, as expected, contained *attL* and *attR* between each construct, the number of which would increase with each round of site-specific integration. The use of nucleases, on the other hand, deletes all SSR footprints, seamlessly connecting gene constructs in the stacked locus.

## Conclusions

We developed a practical approach of transgene stacking that would facilitate rapid introduction of multiple traits into crop varieties without complicating the downstream breeding process. The present work tested each component of the proposed gene stacking method that involves robust SSR systems for directing gene integrations and nucleases for marker excisions. The use of SSR allowed high rates of targeted integrations, which is a major bottleneck in gene targeting. The nucleases were instead used for excising marker genes that involves DSB repair through a more efficient process of joining chromosomal ends. By using the power of each reagent, this study developed an efficient approach of gene stacking that could integrate new genes to the specified site through unlimited rounds of plant transformations.

## Methods

### Plant line and transformation vectors

Transgenic rice line T5 (in variety Taipei 309) that contains a single *lox76* site (right arm mutant) [9] within the construct as depicted in Fig. 2a and described earlier [28] was used as the founder line for gene stacking. Donor vectors, pNS27 and pNS35, for site-specific integration in *lox76* site and *FRT* site, respectively, were constructed in pBluescript SK backbone. pNS27 (Fig. 2b) consists of a promoter-less *Bar* gene flanked by I-*Sce*I target sites (Fig. 3d) followed by a *GFP* gene driven by a 35S promoter as a gene-of-interest, *CCR5* site, the ZFN target, and a promoter-less neomycin phosphotransferase gene (*NPT II*) fused to *FRT* site. The whole construct is flanked by *loxP* upstream and *lox75* downstream. pNS35 (Fig. 4b) contains maize ubiquitin-1 (Ubi) promoter [54] and *GUS* gene flanked by *FRT* sites. Sequences of *lox* and *FRT* are described by Albert et al. [9] and Senecoff et al. [55]. FLPe expression vector, pUbiFLPe, consists of FLPe gene [56] transcribed by Ubi promoter. I-*Sce*I and ZFN expression vectors (pUbiISceI, pUbiZFN, pHSISceI, and pHSZFN) either contained Ubi promoter or soybean heat shock 17.5E (HS) promoter [57]. The optimized I-*Sce*I coding sequence [58] and the *CCR5*-ZFN coding sequence was provided by Drs. Holger Puchta (Karlsruhe, Germany) and Joseph Petolino (Dow Agro Sciences, Inc.), respectively. Each gene in these vectors contain transcription termination sequence of nopaline synthase gene (*nos 3′*).

### Rice transformation

Rice tissue culture media and protocols were essentially as described by Nishimura et al. [59]. All transformations were done by particle bombardment using PDS 1000/He gene gun (Bio-Rad, Inc.) as described earlier [13]. Scutellar calluses generated from mature seeds were bombarded with 1-μm gold particles coated with plasmid DNA. About 25 μg of particles were coated with 5 μg donor vectors (pNS27 or pNS35) or 1 – 2 μg of I-*Sce*I/ZFN vectors and used for ten shots (plates). The pNS27-bombarded T5 callus were selected on bialaphos (5 mg/L) to isolate site-specific integration (SSI) lines. The selected SSI lines were bombarded with I-*Sce*I or ZFN vectors along with a selection vector, pSS1, which contains 35S promoter driven *NPT II* gene, and selected on geneticin (100 mg/L).

### Molecular analysis

Genomic DNA was isolated from the callus or leaves and subjected to PCR using primers (Table 2) and Taq polymerase following the manufacturer’s (Promega, Inc.) recommendations. All PCR reactions consisted of 40 cycles of 1 min denaturation at 94 °C, 1 min annealing at 56 °C and 1 min extension at 72 °C followed by final elongation step for 15 min at 72 °C. For sequencing, the PCR amplicon was extracted from the gel and sequenced by Eurofins MWG Operon Kentucky, and viewed using Sequence Scanner v1.0 (Applied Biosystems). For Southern hybridizations, ~5 μg of genomic DNA was digested overnight with appropriate restriction enzyme, fractionated on 0.8 % agarose gel, transferred to a nylon membrane, and hybridized with P^32^ labelled DNA probes.

**Table 2:** List of primers

Primers	Sequence (5′ → 3′)
a (or a2)	TCTACTTCTGTTCATGTTTGTG
a1	TCTAACCTTGAGTACCTATCTATTA
b	AAGACCCCAACGAGAAGC
c	CTCGATGCGATGTTTCGCTT
d	CTAATCGCCATCTTCCAGCA
e	ACAGGCTGAACTTGTGGC
f	GATGGATTGCACGCAGGTTC
g	GCCACAAGTTCAGCGTGT
ISceI Foward	GCTGTCTCCTCCTCACAAG
ISceI Reverse	GGGTCAGGTAGTTCTCCACC
ZFN Reverse	TGCAGATTCCGACACTGGAAG
Chr 10 locus1 Forward	GCAAGCCGGTCACCATTTTCA
Chr 10 locus1 Reverse	TTCGTTGGTTTGCACGCTAT
Chr 10 locus2 Forward	GAAAGGCGTAACGATCTGGG
Chr 10 locus2 Forward	TAGTACGAGAGGACCGGGAA

## Abbreviations

DSB: Double-stranded break
indels: Insertion-deletion
ZFN: Zinc finger nuclease
SSR: Site-specific recombination
SSI: Site-specific integration
SMG: Selectable marker gene
